# The histidine kinase NahK regulates denitrification and nitric oxide accumulation through RsmA in *Pseudomonas aeruginosa*

**DOI:** 10.1128/jb.00408-24

**Published:** 2024-12-11

**Authors:** Danielle Guercio, Elizabeth Boon

**Affiliations:** 1 Graduate Program in Molecular and Cellular Biology, Stony Brook University 12301 , Stony Brook, New York, USA; 2 Department of Chemistry, Stony Brook University Department of Chemistry 603074 , Stony Brook, New York, USA; 3 Institute of Chemical Biology and Drug Discovery, Stony Brook University 12301 , Stony Brook, New York, USA; University of California San Francisco, San Francisco, California, USA

**Keywords:** nitric oxide, biofilm, NosP, NahK, RsmA, denitrification, quorum sensing, cell elongation

## Abstract

*Pseudomonas aeruginosa* have a versatile metabolism; they can adapt to many stressors, including limited oxygen and nutrient availability. This versatility is especially important within a biofilm where multiple microenvironments are present. As a facultative anaerobe, *P. aeruginosa* can survive under anaerobic conditions utilizing denitrification. This process produces nitric oxide (NO) which has been shown to result in cell elongation. However, the molecular mechanism underlying this phenotype is poorly understood. Our laboratory has previously shown that NosP is a NO-sensitive hemoprotein that works with the histidine kinase NahK to regulate biofilm formation in *P. aeruginosa*. In this study, we identify NahK as a novel regulator of denitrification under anaerobic conditions. Under anaerobic conditions, deletion of *nahK* leads to a reduction of growth coupled with reduced transcriptional expression and activity of the denitrification reductases. Furthermore, during stationary phase under anaerobic conditions, Δ*nahK* does not exhibit cell elongation, which is characteristic of *P. aeruginosa*. We determine the loss of cell elongation is due to changes in NO accumulation in Δ*nahK*. We further provide evidence that NahK may regulate denitrification through modification of RsmA levels.

**IMPORTANCE:**

*Pseudomonas aeruginosa* is an opportunistic multi-drug resistance pathogen that is associated with hospital-acquired infections. *P. aeruginosa* is highly virulent, in part due to its versatile metabolism and ability to form biofilms. Therefore, better understanding of the molecular mechanisms that regulate these processes should lead to new therapeutics to treat *P. aeruginosa* infections. The histidine kinase NahK has been previously shown to be involved in both nitric oxide (NO) signaling and quorum sensing through RsmA. The data presented here demonstrate that NahK is responsive to NO produced during denitrification to regulate cell morphology. Understanding the role of NahK in metabolism under anaerobic conditions has larger implications in determining its role in a heterogeneous metabolic environment such as a biofilm.

## INTRODUCTION

The opportunistic pathogen *Pseudomonas aeruginosa* is a major contributor to ventilator-associated pneumonia, catheter-associated urinary tract infections, and burn wound infections (1–3). In particular, cystic fibrosis (CF) patients are highly susceptible to *P. aeruginosa* lung infections. *P. aeruginosa* thrive within the thick microaerobic mucus layers in the lungs of CF patients, resulting in chronic infections and damage to epithelial tissue (4). *P. aeruginosa* are facultative aerobes; under anaerobic conditions, they utilize nitrate (NO_3_^-^) or nitrite (NO_2_^-^) as alternative electron acceptors in a process termed denitrification (5). During denitrification, nitrate is reduced to nitrogen through the sequential activity of the reductases nitrate reductase (NAR), nitrite reductase (NIR), nitric oxide (NO) reductase (NOR), and nitrous oxide reductase (NOS) (6), thus supporting growth in the absence of oxygen.

Under anaerobic conditions, the principal transcriptional regulator for genes associated with denitrification is arginine deiminase and nitrate reduction regulatory protein (ANR). Under low oxygen conditions, ANR upregulates genes associated with anaerobic metabolism including the transpiration factor dissimilative nitrate respiration regulator (DNR). DNR then promotes the transcriptional expression of the denitrification reductases and is additionally activated by NO, an intermediate in the denitrification process produced by NIR activity (7). This is hypothesized to be a mechanism to prevent accumulation of toxic levels of NO within the cell during denitrification (8). This is important because at high concentrations, NO disrupts iron-sulfur clusters and other iron-binding proteins, nitrosylates cysteines, and causes lipid peroxidation and DNA strand breaks, leading to changes in cell membrane permeability and membrane potential and cell death (9, 10).

In addition to being regulated by NO concentrations through DNR, denitrification reductases are transcriptionally regulated by the *las*, *rhl*, and *pqs* quorum sensing networks (11, 12). The *las* and *rhl* systems transcriptionally downregulate the denitrification reductases; strains that lack either of the response regulators *lasR* or *rhlR* have been shown to have an increase in denitrification activity due to increased expression of the reductases (11). In PAO1 Δ*rhlR,* increased expression of NAR and NIR contribute to an overproduction of NO, leading to cell death in anaerobic biofilms (13). Furthermore, the quorum sensing autoinducer PQS has been shown to reduce anaerobic growth by directly inhibiting the activity of the denitrification reductases NAR, NOR, and NOS through active-site iron chelation. In contrast, PQS promotes the activity of NIR (12). However, in fully anaerobic conditions, PQS is not produced, because its production relies on the monooxygenase PqsH, suggesting that its role in denitrification may only be relevant in microaerobic conditions or the transition between aerobic and anaerobic respiration (11, 12, 14).

NO is well documented as a signal for biofilm dispersal. In *P. aeruginosa,* the NO sensing hemoprotein NosP has been shown to be necessary for NO-mediated biofilm dispersal by inhibiting a co-cistronic histidine kinase NahK (15, 16). NahK is part of the GacS multi-kinase network (MKN) (17). NahK transfers phosphate to the histidine-containing phosphotransfer protein HptB (18). When HptB is unphosphorylated, it plays an indirect role in positively regulating transcription of the small regulatory RNA *rsmY* (19). *rsmY* inhibits translation of the RNA-binding protein RsmA, a post-transcriptional regulator of 100 s of genes, which is thought to be the master switch between biofilm and planktonic growth, promoting bacterial motility and inhibiting quorum sensing and biofilm formation (20).

Recently, we reported that a *nahK* deletion strain dramatically overproduces the molecule pyocyanin through mis-regulation of the PQS quorum sensing system (21). Pyocyanin is a redox active small molecule secreted by *P. aeruginosa* to act as an electron shuttle supporting aerobic respiration under the microaerobic conditions in a biofilm (22, 23). NahK is not alone as a regulator of PQS in the GacS MKN; it has also been reported that Δ*rsmA*, Δ*gacS*, and Δ*retS* overproduce pyocyanin, and Δ*rsmY* and Δ*rsmZ* underproduce pyocyanin, relative to the wild-type strain (19, 24). The overproduction of pyocyanin and mis-regulation of quorum sensing in Δ*nahK* (21), as well as its role in NO-mediated biofilm regulation (15), led us to hypothesize that NahK may play a regulatory role in denitrification, as both NO and pyocyanin are essential for survival in microaerobic conditions. Here, we describe a novel role for NahK in promoting anaerobic biofilm formation and cell elongation through regulation of intracellular NO accumulation.

## RESULTS

### Deletion of *nahK* leads to changes in anaerobic growth

**Fig 1:**
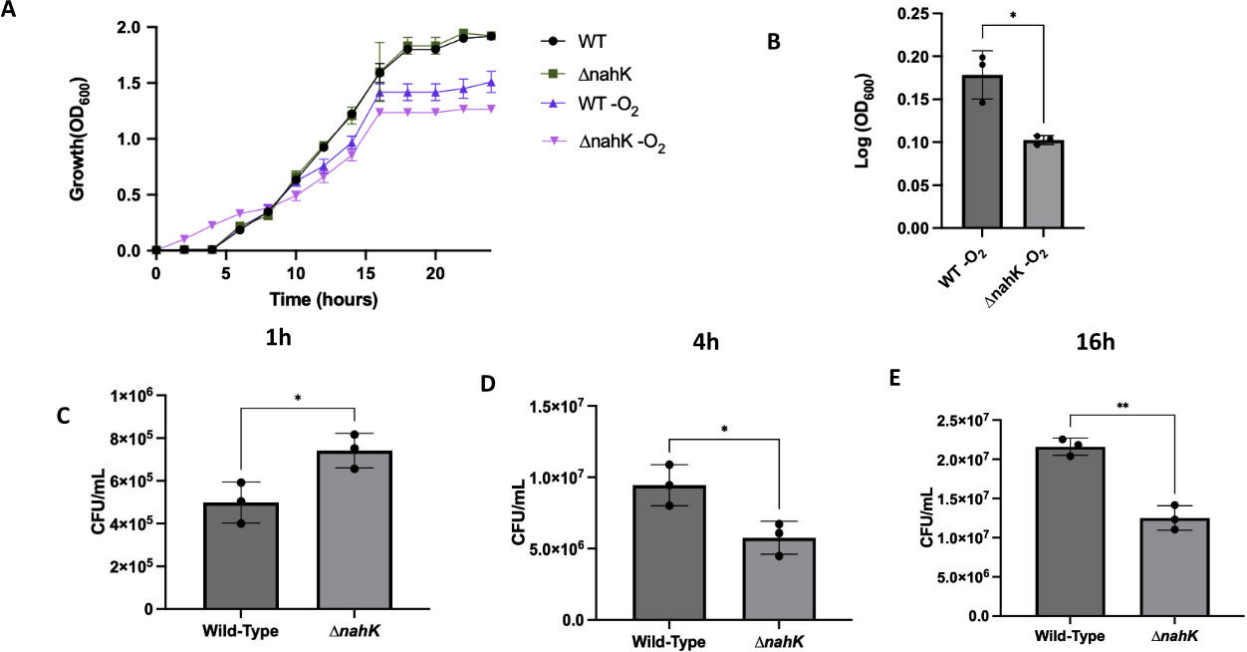
Anaerobic growth is altered in Δ*nahK*. (**A, B**) Cultures with a starting OD of 0.005 were grown in Luria-Bertani (LB) media in the presence of oxygen, or in the absence of oxygen but in media supplemented with 25 mM nitrate (NaNO_3_). OD readings were measured as a function of time. (**A**) The average OD readings, ±1 standard deviation, from three independent biological replicates, are plotted as a function of time. (**B**) Log (OD_600_) at the 24-h time point was quantified in the anaerobic cultures. *P*-values were calculated using unpaired two-tailed *t*-test comparing Δ*nahK* log (OD_600_) to wild-type log (OD_600_); *P ≤ 0.05. (**C–E**) CFU values were calculated after 1 h (**C**), 4 h (**D**), or 16 h (**E**) of anaerobic growth (from the same culture) in LB media supplemented with 25 mM NaNO_3_. The plotted values represent the average CFUs, ± 1 standard deviation, from three independent biological replicates. *P*-values were calculated using unpaired, two-tailed *t*-test comparing Δ*nahK* CFU to wild-type CFU; *P ≤ 0.05, **P ≤ 0.01.

To investigate if NahK has a role in regulation of respiration under microaerobic or anaerobic conditions, first, we investigated the role of NahK in growth under anaerobic denitrifying (addition of nitrate, NaNO_3_) conditions in comparison to a wild-type strain (Fig. 1A). To confirm conditions were indeed anaerobic, we grew cultures without nitrate and observed minimal to no growth (Fig. S1). Under anaerobic conditions, Δ*nahK* initially grows faster in early exponential phase; after 1 h of anaerobic growth, the colony formation units (CFU) value of Δ*nahK* is higher than that of the wild-type strain (Fig. 1B). However, over time, the wild-type strain catches up; the CFUs after 4 h of anaerobic growth and during stationary phase are lower in Δ*nahK* in comparison to wild-type (Fig. 1C, D, and E). Aerobically, we did not observe any differences in CFU counts (Fig. S2). These results suggest that *nahK* alters anaerobic growth and may be required for wild-type growth under these conditions.

### Denitrification reductases are differentially expressed during exponential and stationary phase

**Fig 2:**
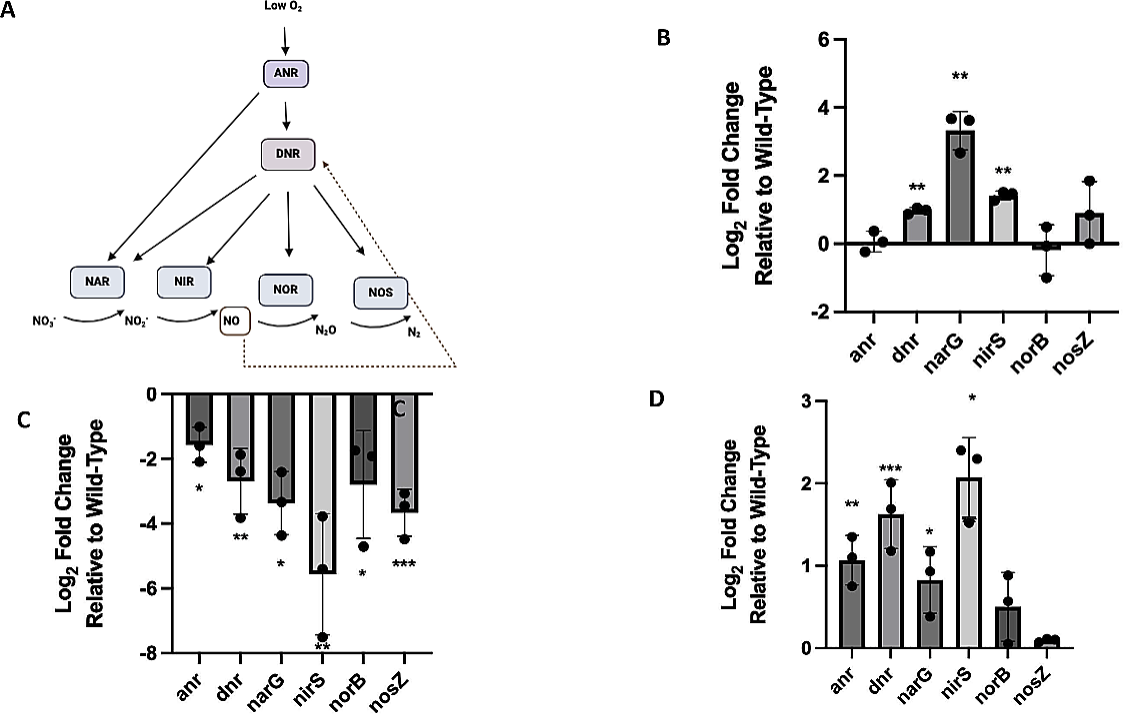
Transcription of the denitrification machinery is mis-regulated in Δ*nahK*. (**A**) Schematic of denitrification in *P. aeruginosa*. (**B–D**) Log_2_ of transcript levels in Δ*nahK*, relative to wild-type, as measured by qPCR, for the four denitrification reductases and two transcriptional regulators are plotted. Gyrase A (*gyrA*) was used as a housekeeping gene; *gyrA* Ct values were similar in aerobic and anaerobic growth in these experiments (Table S4). Cultures were grown in Luria-Bertani media in the presence of oxygen, or in the absence of oxygen but in media supplemented with 25 mM NaNO_3_. RNA was extracted at several time points and growth conditions: (**B**) after 4 h anaerobic growth (exponential stage); (**C**) after 16 h anaerobic growth (early stationary phase); (**D**) and at an OD of 1.0 during aerobic growth. Bars above and below the threshold represent up- and down-regulation in Δ*nahK*, relative to wild-type, respectively. The values represent the average ± 1 standard deviation of three independent biological replicates (each biological replicate consists of two technical replicates). *P-*values were calculated using unpaired, two-tailed *t*-tests comparing Δ*nahK* ΔCt values to wild-type ΔCt values; *n* = 3; *P ≤ 0.05, **P ≤ 0.01, ***P ≤ 0.005.

To understand why we observed a growth difference in Δ*nahK* under anaerobic conditions, we conducted quantitative PCR (qPCR) on the reductase transcripts, as well as the transcripts of the denitrification regulators *anr* and *dnr*. (Fig. 2A). During exponential phase (after 4 h), we observed an up-regulation in transcription of both *dnr* and the reductases in Δ*nahK*, relative to wild type, which could explain the increase in initial growth (Fig. 2B). Furthermore, during stationary phase (after 16 h), we observed a decrease in expression of the reductases, as well as *anr* and *dnr* (Fig. 2C), in Δ*nahK* relative to wild type, which could account for the observed reduced growth after early exponential phase.

Because the increase in reductase expression in Δ*nahK* was observed shortly after the culture was inoculated, we next wanted to see if increased expression of the reductases also occurs under aerobic conditions. Under aerobic conditions, there is an increase in expression of *anr*, *dnr, narG*, and *nirS* (Fig. 2D) in Δ*nahK*, relative to the wild-type strain. This increased expression could indicate that the anaerobic respiration machinery is mis-regulated in Δ*nahK* and the bacteria are behaving as if they are under microaerobic conditions even during aerobic respiration, and thus in our experiments, at the time of inoculation, were primed for anaerobic respiration. This is consistent with inhibition of the *las* and *rhl* quorum sensing systems in Δ*nahK*, as we have previously reported (11).

### Reductase activity is reduced in Δ*nahK*

Since we observed a large difference in expression of the reductases NAR and NIR in Δ*nahK*, relative to the wild-type strain, during both exponential and stationary phase, we wanted to determine if we could measure differences in denitrifying enzyme activities under these conditions. To accomplish this, we quantified nitrite production (from nitrate), nitrite utilization, and NO accumulation, each as a function of time, as readouts for the activity of the reductases NAR and NIR.

**Fig 3:**
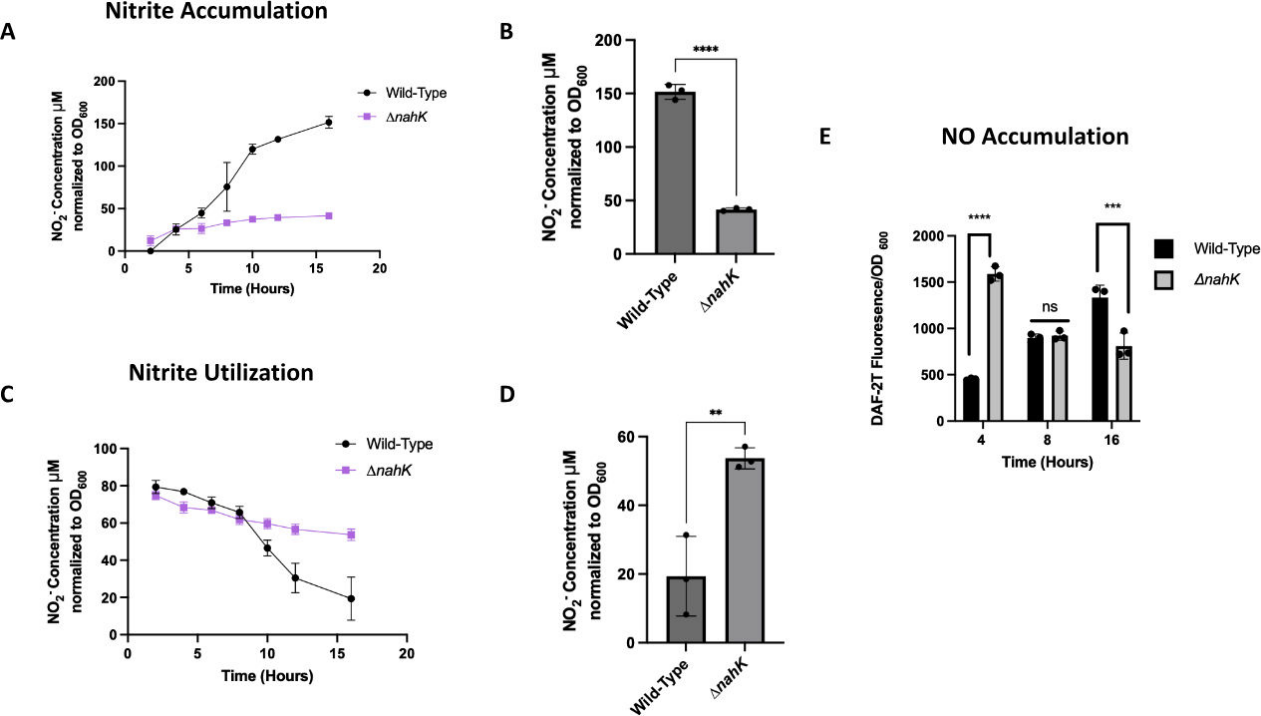
Denitrification activity is mis-regulated in Δ*nahK*. (**A, B**) Nitrite production by NAR during anaerobic denitrifying conditions as a function of time. (**A**) Cultures with a starting OD of 0.005 were grown in Luria-Bertani (LB) media supplemented with 25 mM NaNO_3_. At each time point, the absorbance from the Griess reagent was normalized to the OD_600_ and that value was used to calculate NO_2_^-^ concentration using a NO_2_^-^ calibration curve. The average nitrite concentration, ± 1 standard deviation, from three independent biological replicates (each biological replicate consists of three technical replicates) is plotted as a function of time. (**B**) The average nitrite concentration after 16 h of anaerobic growth (stationary phase), ± 1 standard deviation, is plotted. *P*-values were calculated using unpaired, two-tailed *t*-test comparing Δ*nahK* to wild-type; ****P ≤ 0.001. Initially, Δ*nahK* produces a little more nitrite than wild-type, but over time, nitrite production stagnates in the mutant while steadily increasing in the wild-type. (**C, D**) Nitrite utilization by NIR during anaerobic denitrifying conditions as a function of time. (**C**) Cultures with a starting OD of 0.005 were grown in LB media supplemented with 25 mM NaNO_2_. At each time point, the absorbance from the Griess reagent was normalized to the OD_600_ and that value was used to calculate the amount of NO_2_^-^ remaining in the culture using a NO_2_^-^ calibration curve. The average nitrite concentration, ± 1 standard deviation, from three independent biological replicates (each biological replicate consists of three technical replicates) is plotted as a function of time. (**D**) The average nitrite concentration after 16 h of anaerobic growth (stationary phase), ± 1 standard deviation, is plotted. *P*-values were calculated using unpaired, two-tailed *t*-test comparing Δ*nahK* to wild-type; **P ≤ 0.01. Initially, Δ*nahK* utilizes more nitrite than wild-type, but over time, nitrite utilization stagnates in the mutant while steadily continuing in the wild-type. (**E**) NO produced by NIR during anaerobic denitrifying conditions as a function of time. Cultures with a starting OD of 0.005 were grown in LB media supplemented with 25 mM NaNO_3_. NO concentration was measured using an NO-sensitive fluorescent dye. Fluorescence was normalized to OD_600_ at each time point. The average value, ± 1 standard deviation, is plotted. *P*-values were calculated using unpaired, two-tailed *t*-test comparing Δ*nahK* to wild-type; ***P ≤ 0.005, ****P ≤ 0.001. We observe initial accumulation of NO at early exponential phase (4 h) and then dissipation by stationary phase (16 h) in Δ*nahK*, while in contrast, NO is steadily produced in the wild-type strain.

NAR is responsible for the reduction of nitrate into nitrite, therefore measuring nitrite production was used as a readout for NAR activity. To measure nitrate reduction, we supplemented cultures with nitrate (NaNO_3_) and measured nitrite production (Fig. 3A and B). Nitrite is steadily produced during the entire time course in the wild-type strain. However, after 4 h of growth, we observed very little production of nitrite in Δ*nahK* (Fig. 3A and B). In the wild-type strain, nitrogen species will accumulate in the cell until late stationary phase (after 16 h) when the levels are all decreasing (25).

To measure the activity of NIR, we added nitrite (NaNO_2_) to rich media under anoxic conditions, rather than nitrate, to bypass NAR. Under the conditions of this experiment, although Δ*nahK* has an early advantage, over the time course of the experiment, the wild-type strain utilizes nitrite faster than Δ*nahK* (Fig. 3C and D), indicating greater activity of NIR. Of note, the Δ*nahK* strain does not grow as well as the wild-type strain under these conditions (Fig. S3), thus nitrite utilization levels were normalized to OD. The slow utilization of nitrite in Δ*nahK* cultures after 8 h (Fig. 3C) may be related to the fact that we see a more significant decrease in *nirS* expression in Δ*nahK*, relative to wild type, than any other reductase between 4 h and 16 h of anaerobic growth (Fig. 2B and C).

We also measured NO accumulation as an additional measurement of NIR activity, as NIR converts nitrite to NO. The presence of NO was measured using an NO detection reagent called DAF-2 DA (24). As expected, in Δ*nahK*, there is an initial accumulation of NO in early exponential phase; however, over time, the amount of NO measured in the Δ*nahK* strain is significantly reduced (Fig. 3E). By contrast, in the wild-type strain, NO accumulates throughout the course of the experiment. This reduction in NO produced by Δ*nahK* is consistent with a reduction in NIR activity and an overall lack of anaerobic respiration after early exponential phase.

All these data relating to reductase activity, taken together with our observed down-regulation of reductase expression (Fig. 2) in Δ*nahK*, leads us to conclude sustained denitrification is deficient in this strain. This suggests that NahK may be regulating denitrification under anaerobic conditions in the wild-type strain. We hypothesize this may be a mechanism to reduce the accumulation of toxic NO in *P. aeruginosa* (25).

### *nahK* is required for cell elongation under anaerobic conditions

At high concentrations (~ µM-mM), NO is toxic; bacteria that utilize denitrification have evolved methods to limit NO production and convert it to less toxic species (9, 10). During anaerobic respiration, *P. aeruginosa* undergoes a dramatic morphological change and becomes highly elongated (at least double the length), compared to aerobically grown bacteria (26). Cell elongation refers to bacteria that replicate DNA but do not complete binary fission resulting in a chain of cells. Elongation has been attributed to accumulated NO, for the purpose of diluting intracellular NO concentrations (8, 26). Furthermore, elongation is required for robust biofilm formation under denitrifying conditions (26). Consistent with this, PAO1 Δ*nir* mutants are unable to elongate or form biofilms under anaerobic conditions (24, 26).

**Fig 4:**
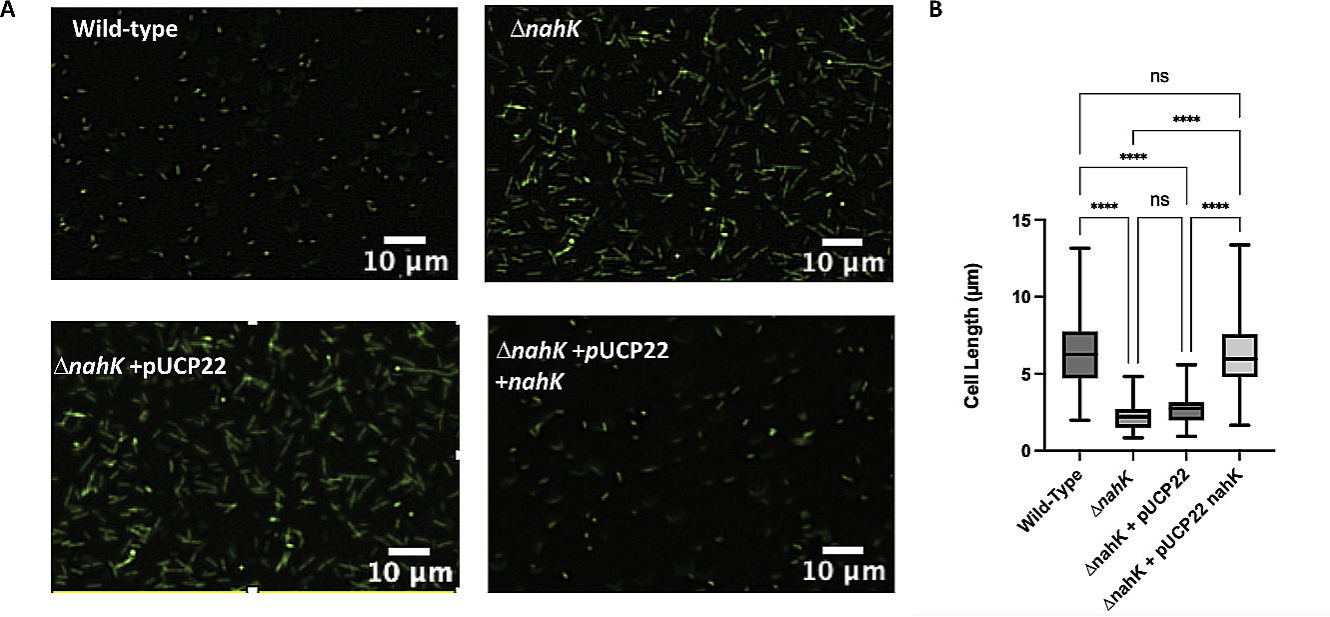
Δ*nahK* prematurely elongates during early exponential phase, in comparison with the wild-type strain. (**A**) Micrographs of wild-type and Δ*nahK*, with and without plasmid-based expression of *nahK*, after 4 h of anaerobic growth in Luria-Bertani, supplemented with 25 mM NaNO_3_ are shown. Cells were fixed in 4% paraformaldehyde, stained with Syto9, and pipetted onto a microscope slide. Three independent cultures (biological replicates) were imaged in three to five random locations; representative images are shown. The premature elongation in Δ*nahK* is consistent with an initial increase in denitrification activity and NO accumulation. This phenotype reverts when Δ*nahK* is transformed with pUCP22 expressing *nahK*, but not empty pUCP22. (**B**) Quantification of cell length. The center line denotes the median cell length (50th percentile), the box contains the 25th to 75th percentiles of measured cell length, and the whiskers mark the maximum and minimum values measured. The select line tool was used to determine the cell length from scaled images using ImageJ. *n* = 75; *P*-values were calculated using one-way analysis of variance and a Tukey multiple comparisons test; ****P ≤ 0.001.

**Fig 5:**
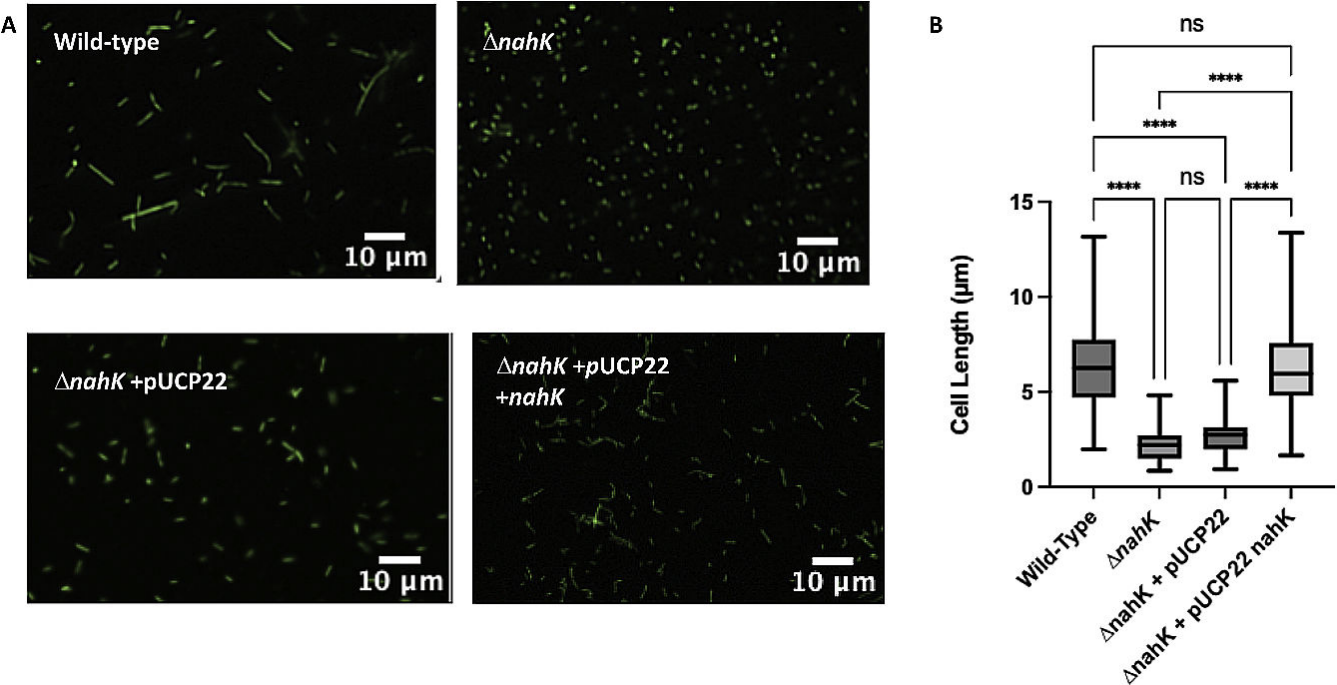
Δ*nahK* is not elongated during stationary phase, unlike the wild-type strain. (**A**) Micrographs of wild-type and Δ*nahK*, with and without plasmid-based expression of *nahK*, after 16 h of anaerobic growth in Luria-Bertani, supplemented with 25 mM NaNO_3_ are shown. Cells were fixed in 4% paraformaldehyde, stained with Syto9, and pipetted onto a microscope slide. Three independent cultures (biological replicates) were imaged in three to five random locations; representative images are shown. The wild-type strain elongates under these conditions; however, the early elongation observed in Δ*nahK* is absent at 16 h, suggesting that denitrification activity and NO accumulation is reduced in this mutant. Elongation is partially recovered when Δ*nahK* is transformed with pUCP22 expressing *nahK*, but not empty pUCP22. (**B**) Quantification of cell length. The center line denotes the median cell length (50th percentile), the box contains the 25th to 75th percentiles of measured cell length, and the whiskers mark the maximum and minimum values measured. The select line tool was used to determine the cell length from scaled images using ImageJ. *n* = 75; *P*-values were calculated using one-way analysis of variance and a Tukey multiple comparisons test; ****P ≤ 0.001.

To assess if changes in NO levels influence elongation in Δ*nahK*, we quantified cell elongation at several time points. Consistent with previous reports, we observed wild-type elongation under anaerobic conditions (Fig. 4 and 5). In contrast, Δ*nahK* initially elongates (after 4 h), but then reverts to normal cell morphology by stationary phase (16 h). This phenotype reverts when Δ*nahK* is transformed with a plasmid expressing *nahK*, but not an empty plasmid. These observations are consistent with the NO concentrations we observed in these strains (Fig. 3).

This difference in elongation at 4 h may also explain why we observe an apparent discrepancy between OD_600_ and CFU measurements of cell growth for Δ*nahK* and the wild-type strain at 4 h (Fig. 1). As illustrated in Fig. 1, the CFUs after 1 h are consistent with the OD_600_-based growth curve; Δ*nahK* is initially growing faster than the wild-type strain. At 4 h, both the OD_600_ and CFU measurements indicate that the Δ*nahK* strain is continuing to grow. However, the wild-type strain appears to be growing more slowly than Δ*nahK* in the OD_600_ measurement (Fig. 1A), but more quickly than the Δ*nahK* strain in the CFU measurement (Fig. 1D). It is possible this discrepancy is because at 4 h, Δ*nahK*, but not wild type, is starting to elongate. The increased cell length in Δ*nahK* could contribute to increased light scattering and a higher OD_600_ reading, without increasing the CFU count. This phenomenon has been reported previously (27) for *Synechococcus elongatus*; the authors reported an increase in OD_600_ and dry weight of the bacteria but no change in CFU.

### Cell elongation is due to differences in NO accumulation

**Fig 6:**
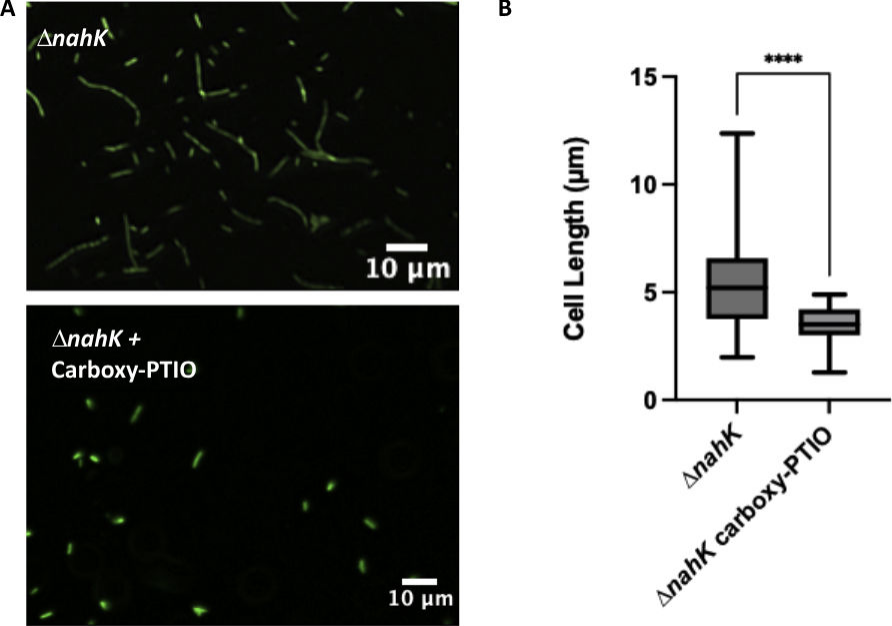
Δ*nahK* does not elongate during early exponential phase in the absence of NO. (**A**) Micrographs of Δ*nahK*, after 4 h of anaerobic growth in Luria-Bertani, supplemented with 25 mM NaNO_3_ and 2 mM carboxy-PTIO (a NO scavenger), are shown. Cells were fixed in 4% paraformaldehyde, stained with Syto9, and pipetted onto a microscope slide. Three independent cultures (biological replicates) were imaged in three to five random locations; representative images are shown. Loss of elongation in Δ*nahK* suggests that elongation during exponential phase is due to accumulation of NO. (**B**) Quantification of cell length. The center line denotes the median cell length (50th percentile), the box contains the 25th to 75th percentiles of measured cell length, and the whiskers mark the maximum and minimum values measured. The select line tool was used to determine the cell length from scaled images using ImageJ. *n* = 75; *P*-values were calculated using an unpaired *t*-test; ****P ≤ 0.001.

To confirm that the changes in elongation in Δ*nahK* are due to differences in NO accumulation over time, Δ*nahK* was grown in the presence of the NO scavenger carboxy-PTIO. After 4 h of growth under anaerobic conditions, Δ*nahK* no longer elongates in the presence of the NO scavenger (Fig. 6). This supports our hypothesis that the early onset of elongation in the deletion strain is due to NO accumulation in early exponential phase (Fig. 6). Likewise, when carboxy-PTIO is added to the wild-type strain, we no longer observe elongation at 16 h (Fig. S4), consistent with our conclusion that NO is driving elongation in our experiments. Furthermore, this result is consistent with similar experiments from other laboratories (8, 25, 26) and serves as a control that addition of carboxy-PTIO is not having unexpected consequences in these experiments.

**Fig 7:**
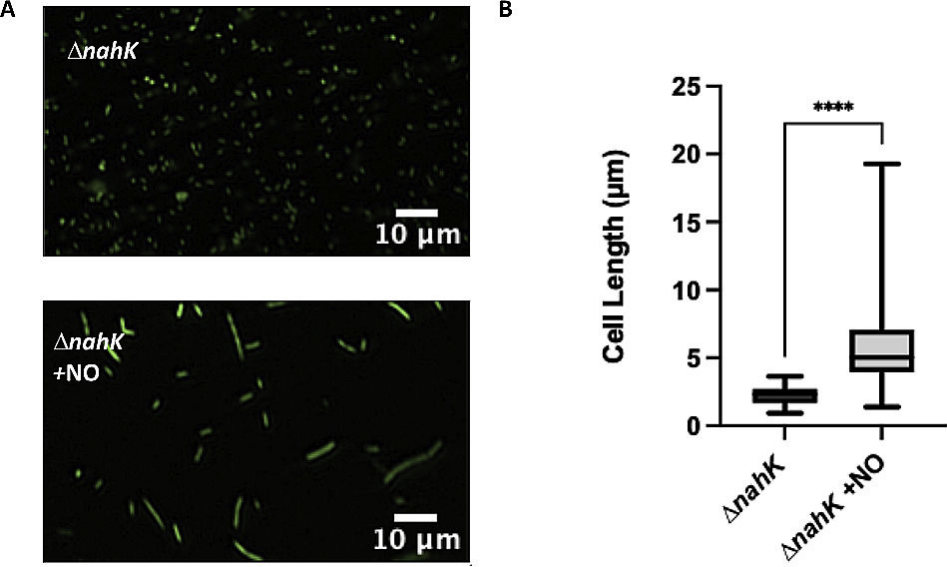
Δ*nahK* elongates during stationary phase anaerobically in the presence of exogenous NO. (**A**) Micrographs of Δ*nahK*, after 16 h of anaerobic growth in Luria-Bertani, supplemented with 25 mM NaNO_3_ and 100 µM DETA-NONOate (a NO donor; ~100 nM NO), are shown. Cells were fixed in 4% paraformaldehyde, stained with Syto9, and pipetted onto a microscope slide. Three independent cultures (biological replicates) were imaged in three to five random locations; representative images are shown. Recovery of elongation in stationary phase in Δ*nahK* in the presence of NO supports our hypothesis that Δ*nahK* does not elongate in stationary phase due to a loss of NO accumulation at this time point. (**B**) Quantification of cell length. The center line denotes the median cell length (50th percentile), the box contains the 25th to 75th percentiles of measured cell length, and the whiskers mark the maximum and minimum values measured. The select line tool was used to determine the cell length from scaled images using ImageJ. *n* = 75; *P*-values were calculated using an unpaired *t*-test; ****P ≤ 0.001.

Likewise, to determine if the lack of cell elongation in Δ*nahK* during stationary phase is due to a reduced amount of NO produced later in growth, we grew both the wild-type and Δ*nahK* strains in the presence of the long-acting NO donor DETA-NONOate until stationary phase. As expected, with the addition of exogenous NO, Δ*nahK* was elongated after 16 h of growth (Fig. 7), but addition of NO has no effect in the wild-type strain (Fig. S5). In wild-type *P. aeruginosa*, although NOR is reducing NO to N_2_O, there is an overall accumulation of NO under anaerobic conditions contributing to the sustained cell elongation over time (26), but addition of extra NO does not have an effect (Fig. S7). These results suggest that the lack of accumulation of NO during stationary phase is contributing to this loss of elongation in Δ*nahK*.

### Loss of NO accumulation and elongation in Δ*nahK* may be due to reduced RsmA levels

NahK is one of several kinases in the GacS multi-kinase network that are important in regulating RsmA (16). RsmA is a post-transcriptional regulator that binds mRNA sequences encoding proteins involved in biofilm formation, thereby preventing their translation and promoting bacteria motility (28). In our previous work, we found that overexpression of *rsmA* in Δ*nahK* restored a pyocyanin overproduction phenotype back to wild-type levels, suggesting a main effect of deletion of *nahK* is the reduction of RsmA levels (21).

**Fig 8:**
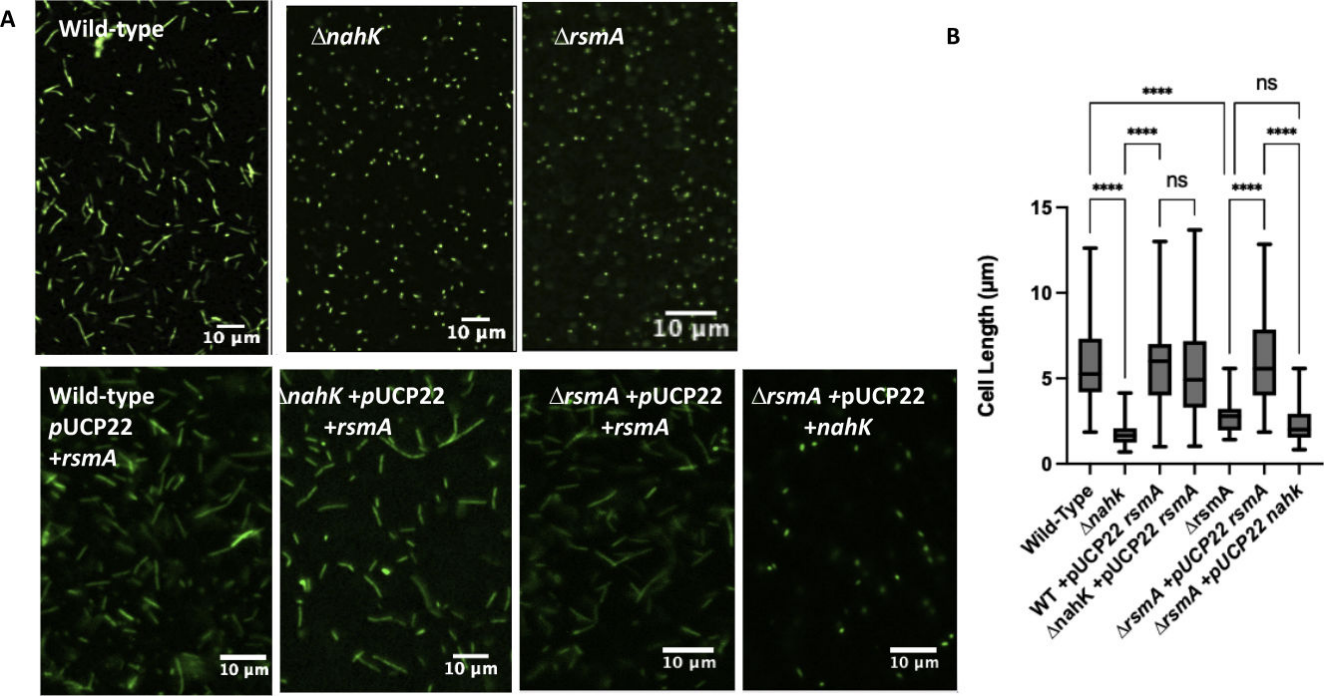
NahK regulates NO-dependent cell elongation through modulation of RsmA levels. (**A**) Micrographs of wild-type, Δ*nahK*, and Δ*rsmA*, with and without plasmid-based expression of *rsmA or nahK*, after 16 h of anaerobic growth in Luria-Bertani, supplemented with 25 mM NaNO_3_, are shown. Cells were fixed in 4% paraformaldehyde, stained with Syto9, and pipetted onto a microscope slide. Three independent cultures (biological replicates) were imaged in three to five random locations; representative images are shown. Overexpression of *rsmA* in Δ*nahK* or Δ*rsmA* restores elongation to wild-type levels. Overexpression of *nahK* in Δ*rsmA* has no effect on the elongation phenotype of Δ*rsmA*. These data suggest that RsmA is downstream of NahK and that RsmA levels are reduced in Δ*nahK*, and that the reduction of NO accumulation in Δ*nahK* is in part due this reduction in RsmA levels. (**B**) Quantification of cell length. The center line denotes the median cell length (50th percentile), the box contains the 25th to 75th percentiles of measured cell length, and the whiskers mark the maximum and minimum values measured. The select line tool was used to determine the cell length from scaled images using ImageJ. *n* = 75; *P*-values were calculated using one-way analysis of variance and a Tukey multiple comparisons test; ****P ≤ 0.001.

To determine if the denitrification phenotypes we observed in this study are due to a similar mechanism, we measured cell elongation in Δ*nahK* expressing *rsmA* during stationary phase (16 h) under anaerobic conditions (Fig. 8). As expected, the overexpression of *rsmA* restored elongation in Δ*nahK,* supporting our previous conclusion that RsmA levels are inhibited in Δ*nahK*. Furthermore, we measured cell elongation in Δ*rsmA* after 16 h and found, as in Δ*nahK*, Δ*rsmA* displays reduced cell elongation (Fig. 8). To confirm that RsmA levels are regulated downstream of NahK, we overexpressed *nahK* in Δ*rsmA* and found it made no difference in cell elongation; this strain phenotypically resembles Δ*nahK* or Δ*rsmA*. As expected, in the exponential stage (4 h) of anaerobic growth, as in Δ*nahK*, we observed increased cell elongation in Δ*rsmA*, and overexpression of *rsmA* in Δ*nahK* restores wild-type growth (Fig. S6). The data presented here suggest a novel role for NahK and the RsmA network in the regulation of denitrification activity and accumulation of NO levels.

### NahK promotes biofilm formation under anaerobic conditions

**Fig 9:**
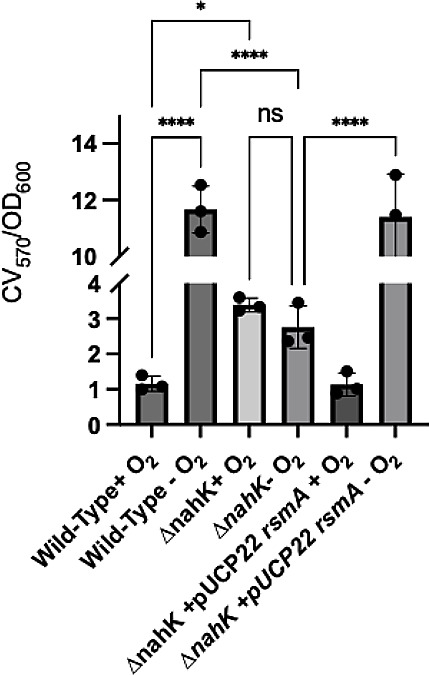
Anaerobic biofilm formation is mis-regulated in Δ*nahK*. A static biofilm assay was utilized to assess biofilm formation in *P. aeruginosa* strains grown 16 h in Luria-Bertani media in the presence of oxygen, or in the absence of oxygen but in media supplemented with 25 mM NaNO_3_. Anoxic incubation was achieved using Thermo Scientific AnaeroPack-Anaero Anaerobic Gas Generator system. Surface attached biofilms were stained using 0.1% crystal violet (CV) solubilized in 30% acetic acid. Biofilm mass was quantified by the absorption of CV (570 nm) normalized to cell optical density (600 nm). Average biofilm mass, ± 1 standard deviation, from three independent biological replicates (each biological replicate consists of three technical replicates) is plotted as a function of time. *P*-values were calculated using one-way analysis of variance and a Tukey multiple comparisons test; *P ≤ 0.05, ****P ≤ 0.001.

Anaerobic cell elongation, presumably due to accumulated NO, has been linked to better anaerobic biofilm formation in *P. aeruginosa* (25). Interestingly, NO is also reported to cause mature biofilm dispersal (15, 16). Nonetheless, because in Δ*nahK* we observe a lack of cell elongation during stationary phase, and a reduction in NO accumulation, we decided to investigate anaerobic biofilm formation in this mutant. For a baseline comparison, we first compared Δ*nahK* and wild-type strains in an aerobic static biofilm assay (Fig. 9). We observed an increase in biofilm formation in Δ*nahK*, compared to the wild-type strain, under these conditions. Under anaerobic conditions, however, we observed a significant increase in wild-type biofilm formation, when compared to biofilm formation under aerobic conditions. This change in biofilm formation as a function of oxygen concentration is not observed in Δ*nahK*. Furthermore, overexpression of *rsmA* in Δ*nahK* restores the mutant to wild-type biofilm formation under both aerobic and anaerobic conditions. We attribute the reduction in anaerobic biofilm formation in Δ*nahK* to the loss of cell elongation and NO accumulation in this mutant (presumably downstream of reduced RsmA levels).

Presumably NahK activity is modulated by the NosP/NO complex in the wild-type, which directs RsmA-mediated biofilm regulation in response to NO generated during denitrification. Interestingly, under aerobic conditions, NO detection triggers biofilm dispersal (15); however, NO-driven cell elongation is required for robust biofilm formation when oxygen is not present (26), thus it may be advantageous for bacteria to prevent NO-mediated biofilm dispersal under anaerobic conditions. Likewise, RsmA levels typically promote bacteria motility; however, these data suggest that under anaerobic conditions, RsmA promotes biofilm formation, presumably due to changes in response to NO accumulation. NahK may contribute to this activity switch. Understanding precisely how and why NO/NosP/NahK contribute to RsmA- and quorum sensing-mediated biofilm regulation in the presence and absence of oxygen is needed to better understand the role of NO in biofilm formation. More experiments will be required to understand this fully.

## DISCUSSION

**Fig 10:**
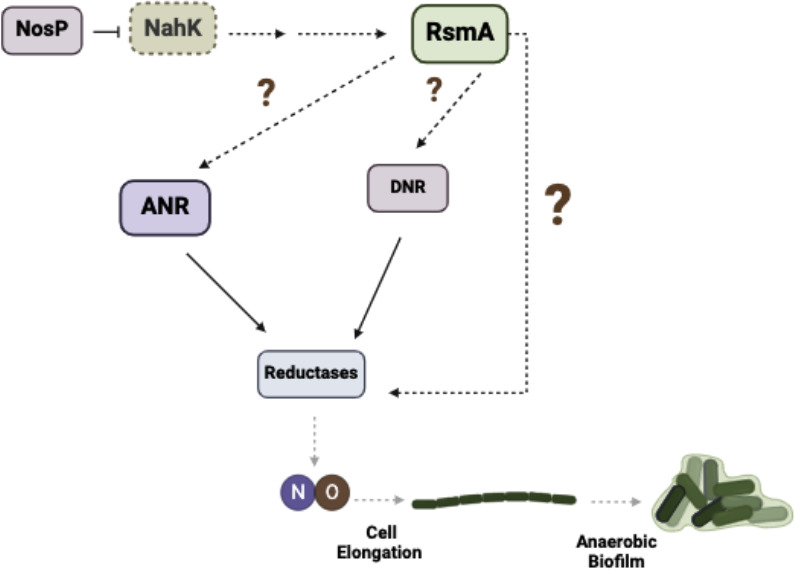
NahK regulates denitrification and anaerobic biofilm formation through the modulation of RsmA levels. Inhibition of NahK contributes to a reduction in RsmA signaling. This reduction reduces the expression of the reductases and reduces denitrification activity. This loss of denitrification activity contributes to reduced levels of NO accumulation resulting in a loss of cell elongation and biofilm formation under anaerobic conditions.

Here, we describe the role of the histidine kinase NahK on NO-dependent regulation of denitrification under anaerobic conditions (Fig. 10). We have shown that loss of *nahK* results in early and unsustainable transcription-driven denitrification activity, along with reduced growth, and loss of NO-driven cell elongation at higher cell densities. Loss of NO-driven cell elongation also results in a reduction of biofilm under oxygen-limited concentrations. Furthermore, we have demonstrated that these phenotypes in Δ*nahK* are driven by reduced RsmA levels: *rsmA* overexpression in the Δ*nahK* mutant restores wild-type cell elongation and biofilm phenotypes, and Δ*rsmA* has the same elongation phenotypes as Δ*nahK*. Overall, our data suggest that NahK activity regulates RsmA levels and that RsmA is required for regulation of denitrification. Therefore, we propose that RsmA may play a role in in the regulation of NO accumulation under anaerobic conditions.

Given that NosP binds NO and regulates NahK (15), it would make sense that NO generated during denitrification is acting as a feedback signal to regulate RsmA through NahK. However, it is clear that NahK activity is not needed for detection of the NO levels that drive cell elongation. The Δ*nahK* strain elongates in response to NO generated at low cell density (Fig. 4) and upon supplementation with exogenous NO at high cell density (Fig. 7). We hypothesize that loss of NahK might cause the cells to be more sensitive to low concentrations of NO than the wild-type strain. This might explain why Δ*nahK* elongates at low cell density, when presumably NO concentration in culture is lower than at higher cell density. Indeed, the amount of NO added to Δ*nahK* that restores cell elongation at high cell density is actually very low (~100 nM), an amount that is insufficient to drive elongation or other stress phenotypes when added to the wild-type strain under aerobic conditions (Fig. S7). Interestingly, when Δ*nahK* is grown aerobically in the presence of NO, we observe slight elongation of some cells, which is not seen in the wild-type strain under these conditions (Fig. S7), but not to the same extent as under denitrifying conditions. This may also be evidence that Δ*nahK* is more sensitive to NO than wild type. Exactly why the cells are more sensitive to NO is not clear at this point.

Nonetheless, there are data in the literature that may help to explain the results we report here. *Burkholderia pseudomallei* can generate intracellular NO through denitrification under low oxygen conditions (29). The presence of nitrate and nitrite has been shown to reduce pellicle biofilm formation in *B. pseudomallei,* both aerobically and anaerobically (30). Furthermore, in the presence of nitrate and nitrite, the *nosP/nahK/narR* operon is upregulated in *B. pseudomallei*, along with several other genes involved in anaerobic metabolism and antibiotic tolerance (28). This up-regulation is dependent on the two-component system NarX/NarL, which is a conserved two-component system in *P. aeruginosa* (31, 32). NarX is sensitive to nitrate and initiates the activation of NarL through autophosphorylation (31, 32). This suggests that NarXL may regulate NosP and NahK in *B. pseudomallei*, and perhaps also in *P. aeruginosa*. Taken together, these studies suggests that detection of nitrate is essential for regulation of metabolism and biofilm formation, possibly through NahK/RsmA-regulated fine tuning of intracellular NO levels. Further studies will be needed to test this mechanism and determine if it is conserved in other facultative aerobic bacteria.

It is surprising that the role of the RsmA network in denitrification remains relatively unexplored in *P. aeruginosa*. As mentioned above, RsmA is regulated by the GacS multi-kinase network, and is thought to serve as the molecular switch between biofilm formation and bacterial motility (20). The GacS network includes GacS, RetS, SagS, PA1611, and NahK, which indirectly regulate RsmA levels through its inhibitory small regulatory RNAs *rsmY* and *rsmZ* (19). Transcript analysis has been thoroughly characterized in Δ*rsmA* (28, 32). In Δ*rsmA*, a decrease in the expression of *anr*, *nirS*, *narG,* and *norC* has been reported (28). Loss of *rsmY* has been previously reported to upregulate azurin, a blue copper protein that supports denitrification by transferring electrons to NIR; azurin is expressed under low oxygen conditions (19, 33, 34). Furthermore, two studies have recently reported that nitrate reductase activity is reduced in Δ*retS* under anaerobic conditions (35–37). RetS is known to inhibit the type IV secretion system gene cluster H2-T6SS. One of the H2-T6SS secreted proteins is ModA, a molybdate scavenger, which is required for anaerobiosis because molybdenum is an important cofactor for NAR (37).

Moreover, RsmA is an inhibitor of *lasR*, which is an inhibitor of denitrification (11, 20). Interestingly, a common mutation in *P. aeruginosa* isolates from CF patients is a deletion of the *lasR* gene, which is thought to promote a survival in anaerobic biofilms in the CF lung (38). This increase in fitness is due to a shift in metabolism in these mutants, in which there is less consumption of oxygen and more utilization of nitrate (37). This shift in metabolism is attributed in part to increased activity of ANR and the denitrification reductases, although the molecular mechanism of the increase in ANR activity is currently unknown (39). The metabolism shift in the Δ*lasR* mutant also promotes fitness by promoting higher antibiotic resistance to tobramycin and ciprofloxacin, which are commonly used to treat *P. aeruginosa* infections in CF patients compared to its LasR+ counterpart (39, 40). Interestingly, these studies lead to the prediction that NahK may have implications in antibiotic resistance. All these observations are important in electron shuttling under microaerobic conditions and the changes in their production allude to a greater role of the GacS multi-kinase network in denitrification regulation. Each of these factors need to be individually characterized to establish the overall mechanism of this system, however.

Although relatively unexplored, there may be an association between NO and the formation of persister cells (8). Persister cells are a subpopulation of metabolically inactive bacteria within a microbial community that are tolerant to harsh conditions, including antibiotics, and help ensure the long-term survival of a biofilm. Biofilms promote the formation of persister cells in part due to the heterogeneity of oxygen and nutrient availability within the biofilm (41). In *Escherichia coli*, NO may inhibit persister cell formation; *E. coli* treated with NO at the onset of stationary phase form significantly fewer persister cells (42). Additionally, transfer of *E. coli* to anaerobic conditions prior to entry into stationary phase reduces persister formation up to 1,000-fold (42, 43). In *P. aeruginosa,* however, NO may increase persister cell formation. NO can activate the SOS response (8), which is one factor that contributes to the formation of persister cells. In the SOS response, RecA de-represses genes involved in DNA repair (44). The activation of RecA also contributes to the formation of membrane vesicles, which are involved in extracellular transport of proteins and quorum sensing signals, in addition to playing a role in antibiotic neutralization (44, 45). It has been reported that *P. aeruginosa* Δ*nirS* is unable to produce membrane vesicles anaerobically, suggesting that production of NO is essential for the activation of SOS and formation of these vesicles (8).

Although the link between NO and persistence is not well established, if NO does trigger persistence in *P. aeruginosa*, it is possible that NahK plays a role in that process. In this study, we demonstrate that cells lacking NahK have mis-regulated denitrification and appear to overreact to NO produced during denitrification, eventually leading to stagnated respiration. In the absence of oxygen and with reduced denitrification activity, we observe reduced growth (Fig. 1), but most of the cells survive in these experiments. It is therefore reasonable to hypothesize the cells may enter a dormant or persistent state.

Evidence for a possible link between NahK and persister cell formation is based on the role of NahK in regulating quorum sensing (16, 21). We have reported that PQS, and several of its precursors, including the molecule 2-AA, are overproduced in the Δ*nahK* strain (21). It was recently published that that 2-AA promotes the formation of persister cells (46). When *mvfR*, the gene that encodes the protein that produces 2-AA, is deleted from *P. aeruginosa*, fewer persister cells are formed than in the wild-type strain. Addition of 2-AA to Δ*mvfR* results in an increase in a ribosomal modulation factor that promotes ribosomal inactivity in persister cells (47). Therefore, it is possible that NahK may play a role in connecting anaerobic respiration, NO sensing, and the production of 2-AA to inform decisions about entering the persistence state.

Antibiotics are commonly used as isolation methods to identify persister populations (47), as persister cells are more resistant to antibiotics than the same cells before entering persistence. Consistent with the Δ*nahK* strain being more prone to formation of persister cells, we have found that Δ*nahK* biofilms are more resistant to ampicillin exposure than wild type (Guercio, D.; Hudson-Smith, N.; and Boon, E.M., unpublished observations). This observation requires additional study to contribute it to persistence, however, because the Δ*nahK* strain also forms more biofilm than wild type under aerobic conditions (Fig. 9), which could be responsible for the increase in antibiotic resistance.

In conclusion, our study implicates NO and NahK in regulating denitrification through RsmA. Understanding how NahK directs denitrification activity may have important implications for how NO sensing through NosP contributes to the molecular mechanisms that regulation oxygen-dependent biofilm formation, and possibly also the development of persistence.

## MATERIALS AND METHODS

### Bacterial strains and growth conditions

Bacterial strains and plasmids used in this study are described in Tables S1 and S2. *P. aeruginosa* strains were grown aerobically in Luria-Bertani (LB) broth. Anaerobic bacterial growth was achieved using Thermo Scientific AnaeroPack-Anaero Anaerobic Gas Generator system or in a Coy vinyl anaerobic chamber. Planktonic bacteria were grown in Hungate anaerobic tubes. The gas composition inside the anaerobic chamber used a mix of nitrogen and hydrogen (95% nitrogen and 5% hydrogen, respectively). To support anaerobic growth, 25 mM NaNO_3_ or 25 mM NaNO_2_ was added to the media. To confirm growth conditions were anaerobic, no detectable growth was observed in LB without NaNO_3_ (Fig. S1). Cultures were grown at 37°C shaking at 250 rpm.

### Growth curve and CFU counting

Day cultures were grown from overnight cultures to an OD_600_ of 1.0 in LB media. Cultures were diluted to an OD_600_ of 0.005 into 5 mL of fresh LB or into 5 mL of fresh LB supplemented with 25 mM NaNO_3_. Cultures were grown either aerobically and anaerobically. Every 2 h, the OD_600_ was measured, and cultures were returned to the incubator. CFU counting was conducted using a previously described method (48).

### qPCR

For qPCR, anaerobic conditions were grown in 5 mL LB media supplemented with 25 mM NaNO_3_ for 4 h (exponential phase) or 16 h (stationary phase). For aerobic conditions, bacteria were grown in 5 mL LB media to an OD_600_ of 1.0. For all conditions, bacteria were grown at 37°C with agitation. Total cellular RNA extraction was performed using the RNeasy Mini Kit (Qiagen). RNA yield was quantified using nanodrop and purity was confirmed by using a 1% agarose gel. cDNA synthesis was performed using RevertAid First Strand cDNA synthesis kit (Thermo Scientific) from purified RNA. The qPCR reaction included 0.3 µM of forward and reverse primers described in Table S3, equal amounts of synthesized cDNA, and 5 µL OF SYBR green master mix (Thermo Scientific) for a total reaction volume of 10 µL. Reaction was performed on a LightCycler 480 with the cycler parameters of 95°C for 10 m, then 40 cycles of 95°C for 15 s and 60°C for 60 s. *gyrA* was used as a housekeeping gene and relative expression was determined using a standard curve (49) (Table S4).

### Denitrification activity assays

Denitrification activities were measured using previously described methods (24) NO_2_^-^ concentration was determined using a Griess Detection Kit (Thermo Scientific). To assess NO_2_^-^ concentration, bacteria were grown anaerobically with a starting OD of 0.005 in LB media supplemented with 25 mM nitrate (NaNO_3_) or 25 mM nitrite (NaNO_2_) to measure NO_2_^-^ production and NO_2_**^-^** utilization over time, respectively. Supernatant was collected from centrifuged cells and incubated with prepared Griess reagent for 30 m. Absorbance was read at 548 nm in reference to the photometric reference sample. At each time point, the absorbance from the Griess reagent was normalized to the OD_600_ and that value was used to calculate the amount of NO_2_^-^ remaining in the culture using a NO_2_^-^ calibration curve.

### NO detection

Cellular NO levels were measured using a previously described method (24) using the NO detection reagent diaminofluorescein-2 diacetate (DAF-2 DA). One milliliter of shaking cultures grown under anaerobic conditions was incubated with 10 µM DAF-2 DA at 7°C for 1 h and washed with phosphate-buffered saline (PBS). Fluorescence of the reaction product DAF-2T was assessed using a SpectraMax iD3 plate reader at an excitation wavelength of 495 nm and emission wavelength of 515 nm normalized to OD_600_.

### Fluorescent microscopy and cell length quantification

Cultures were grown in LB media in the presence of oxygen, or in the absence of oxygen but in media supplemented with 25 mM nitrate (NaNO_3_). For all sample preparations, cells were fixed in 4% paraformaldehyde. The nucleic acid stain SYTO9 green-fluorescent dye was used at a final concentration of 10 µM. A Zeiss Axio Vert.A1 inverted epifluorescence microscope equipped with a Lumencor Sola Light Engine, a Lumenera 8MP Infinity3 Camera, a Zeiss GFP fluorescence filter set to 38 HE, and a Zeiss A-Plan 40× N.A. 0.55 objective was used. The select line tool was used to determine the cell length from scaled images using ImageJ. To assess cell elongation in the presence of exogenous NO, 100 µM of DETA-NONOate was added to LB media supplemented with 25 mM NaNO_3_ where cultures were grown anaerobically for 16 h. To assess cell elongation dependence on the presence of NO, cultures were grown anaerobically in LB media supplemented with 25 mM nitrate (NaNO_3_) and 2 mM of the NO scavenger carboxy-PTIO.

### Static biofilm assay

Ability of wild-type and Δ*nahK* to form biofilm under aerobic and anaerobic conditions was assessed using a modified microtiter dish assay in six-well plates to quantify surfaced attached bacteria grown statically (50). To assess biofilm formation aerobically, 5 mL day cultures were prepared from overnight cultures and grown until the cultures reached an OD_600_ of 1.0. Five hundred microliters of day culture was added to each well which contained 9.5 mL of LB or LB supplemented with 25 mM NaNO_3_. To assess biofilm formation anaerobically, day cultures at an OD_600_ of 1.0 were diluted to an OD_600_ of 0.005 into 5 mL of fresh LB and grown for 16 h. Five hundred microliters of anaerobic overnight culture was added to each well which contained 9.5 mL of LB supplemented with 25 mM NaNO_3_ to achieve anaerobic growth; Thermo Scientific AnaeroPack-Anaero Anaerobic Gas Generator system was used. Six-well plates were incubated at 37°C statically for 16 h. After 16 h, OD_600_ of the planktonic cells was recorded on the SpectraMax iD3 plate reader. The remaining planktonic cells were removed, and the plate was rinsed with ultra-pure water. The plate was dried and stained with 2 mL/well 0.1% crystal violet (CV) for 15 minutes with agitation. Excess CV was rinsed with ultra-pure water. After drying for 1 h, 2 mL 30% acetic acid was added to each well to solubilize the CV. CV was quantified by absorbance and was read at 570 nm to quantity CV. CV570 was normalized to OD_600_ to account for variation in planktonic growth although OD_600_ was consistent from strain to strain in each condition.

## Data Availability

All requests for resources and reagents should be directed to and will be fulfilled by the Lead Contact Elizabeth Boon (elizabeth.boon@stonybrook.edu). All constructs and cell lines generated in this study are freely available upon request.

## SUPPLEMENTAL MATERIAL

Supplemental tables and figures jb.00408-24-s0001.pdf Tables S1 to S4; Fig. S1 to S7.

ASM does not own the copyrights to Supplemental Material that may be linked to, or accessed through, an article. The authors have granted ASM a non-exclusive, world-wide license to publish the Supplemental Material files. Please contact the corresponding author directly for reuse.
