# Supplementary material for: The histidine kinase NahK regulates denitrification and nitric oxide accumulation through RsmA in Pseudomonas aeruginosa
Source: J Bacteriol. 2024 Dec 11;207(1):e00408-24. doi: 10.1128/jb.00408-24 (PMC11784011; doi:10.1128/jb.00408-24)
Supplement: Supplemental tables and figures — Tables S1 to S4; Fig. S1 to S7. [file jb.00408-24-s0001.pdf]

**Supplemental Materials:**

# The Histidine Kinase NahK Regulates Denitrification and Nitric Oxide Accumulation through RsmA in *Pseudomonas aeruginosa*

Danielle Guercio[1], Elizabeth Boon[1,2,3,#]

(1) Graduate Program in Molecular and Cellular Biology; (2) Department of Chemistry; (3) Institute of Chemical Biology and Drug Discovery; Stony Brook University, Stony Brook, NY; # Corresponding author: Elizabeth M. Boon elizabeth.boon@stonybrook.edu

**Table S1. Strains used in this study.**

| Strains | Relevant Characteristics | Source |
|---|---|---|
| ***P. aeruginosa*** | | |
| UCBPP-PA14 | Wildtype | Lab Collection |
| PA14 Δ*nahK* | PA14 deletion mutant for PA14_38970 (*nahK*). | Lab Collection (20) |
| PA14 Δ*rsmA* | PA14 deletion mutant for PA14_52570 | Generous gift from Lars Deitrich |

**Table S2. Plasmids used in this study.**

| Plasmid | Relevant Characteristic | Source |
|---|---|---|
| pUCP22 | $Amp^R$ $Gen^R$ | Addgene |
| pUCP22 *nahK* | $Amp^R$ $Gen^R$ containing *nahK* gene. | Lab Collection (20) |
| pUCP22 rsmA | $Amp^R$ $Gen^R$ containing *rsmA* gene. | Lab Collection (20) |

**Table S3. Primers used in this study.**

| Primer | Oligonucleotide |
|---|---|
| qPCR gyrA fwd | TGTGCTTTATGCCATGAGCGA |
| qPCR gyrA rev | TCCACCGAACCGAAGTTGC |
| qPCR anr fwd | CAACGAGATCGGCAACTATC |
| qPCR anr fwd | GCACTTCCTTGCCTTCC |
| qPCR dnr fwd | CGCACGCCTTCTACTACCTG |
| qPCR dnr rev | GAAAGCGTCTCGATCTCGTC |
| qPCR narGfwd | GAGCTTCTACGACTGGTACT |
| qPCR narG rev | CGATGATGTAGCTGGAGTTG |
| qPCR nirS fwd | CTGTTCATCAAGACCCATCC |
| qPCR nirS rev | GGTTCTTCAGGTCGAACAC |
| qPCR norB fwd | CTGTTCCTGTTCTCCTTCTAC |
| qPCR norB rev | CCATGATCAGTTCCCACAC |
| qPCR nosZ fwd | CTCAGCAAGTTCTCCAAGG |
| qPCR nosZ rev | CGTGGACCAGCTTCATTT |

**Table S4. Ct values of housekeeping gene *gyrA***

| **wild-type aerobic** | **∆*nahK* aerobic** | **wild-type 4h anaerobic** | **∆*nahK* 4 h anaerobic** | **wild-type 16 h anaerobic** | **∆*nahK* 16 h anaerobic** |
|---|---|---|---|---|---|
| 15.18 | 14.99 | 16.13 | 15.72 | 16.23 | 16.22 |
| 14.81 | 15.23 | 16.02 | 15.85 | 15.69 | 15.78 |
| 15.17 | 15.10 | 15.98 | 15.86 | 15.61 | 15.83 |

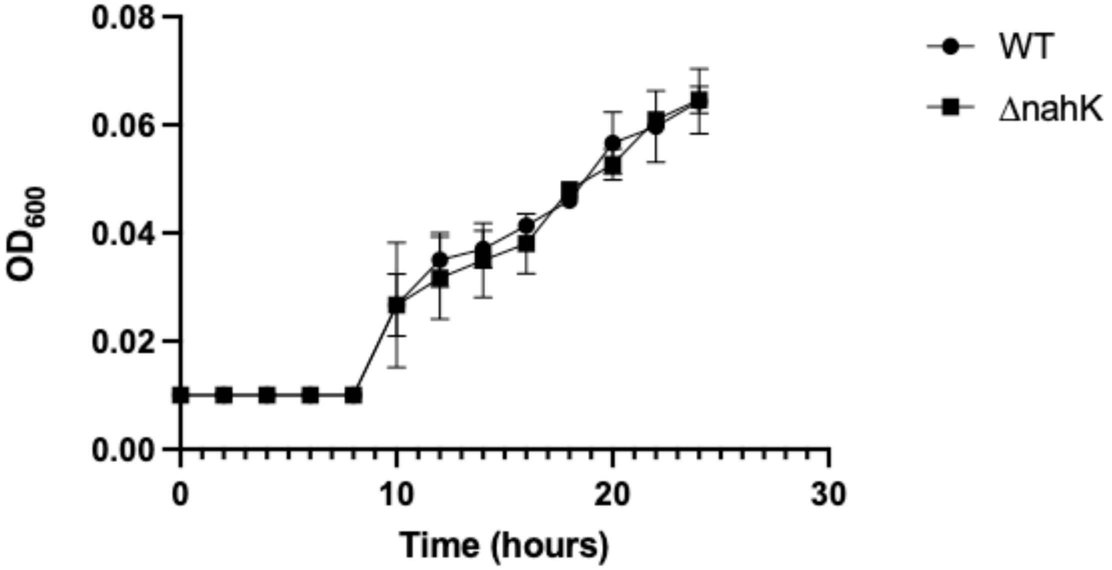


**Figure S1. *P. aeruginosa* wild-type and ∆*nahK* strains do not grow well under anaerobic conditions in the absence of nitrate.** Cultures with a starting OD of 0.005 were grown in LB media in absence of oxygen with no addition of nitrate or nitrite. OD readings were measured as a function of time. The average OD readings, ± 1 standard deviation, from 3 independent biological replicates, are plotted as a function of time.

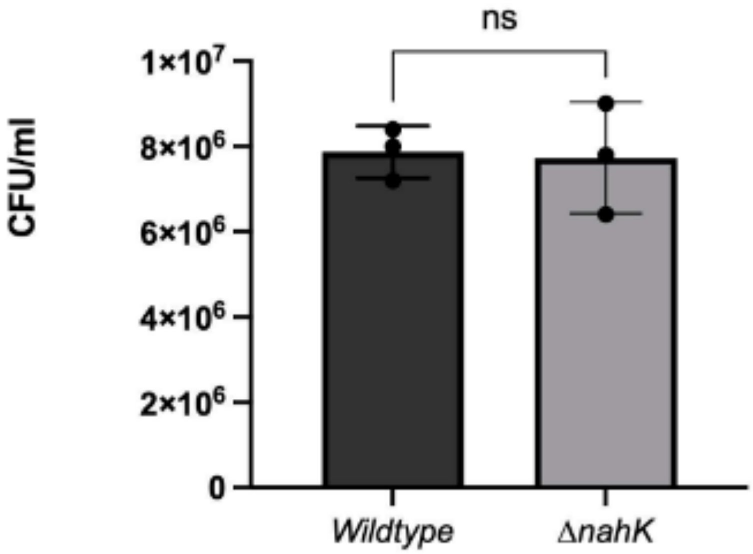


**Figure S2. *P. aeruginosa* wild-type and ∆*nahK* strains grow equally well in aerobic shaking conditions.** CFU values form an aerobic shaking culture in LB media grown to an OD of 1.0 were calculated. The plotted values represent the average CFUs, ± 1 standard deviation, from 3 independent biological replicates. *p*-values were calculated using unpaired, t-tailed t-test comparing ∆*nahK* OD to wild-type OD; $p > 0.05$.

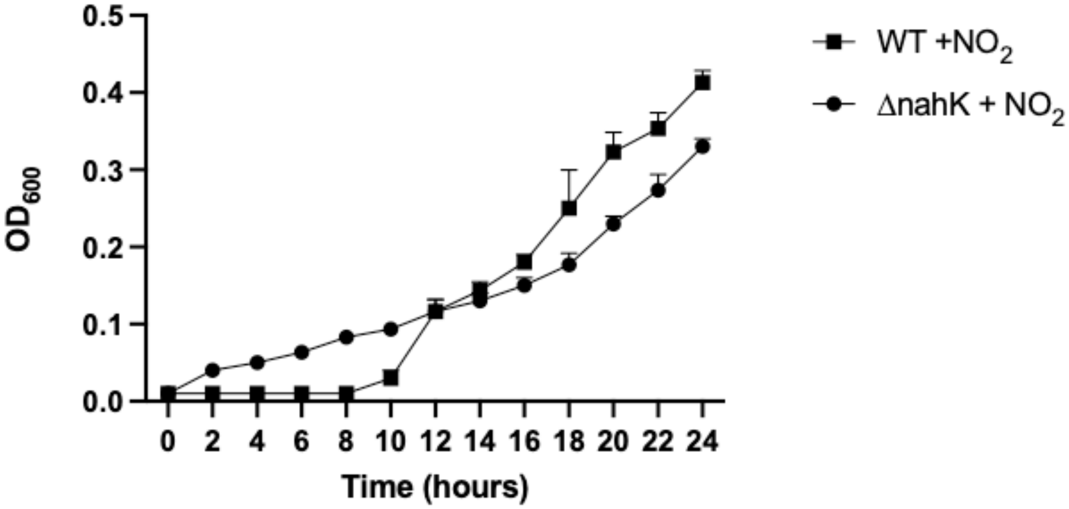


**Figure S3. The Δ*nahK* strain has altered growth in anaerobic culture in the presence of nitrite, in comparison to the wild-type strain.** Cultures with a starting OD of 0.005 were grown in LB media in the absence of oxygen but in media supplemented with 25 mM nitrite ($NaNO_2$). OD readings were measured as a function of time. The average OD readings, ± 1 standard deviation, from 3 independent biological replicates, are plotted as a function of time.

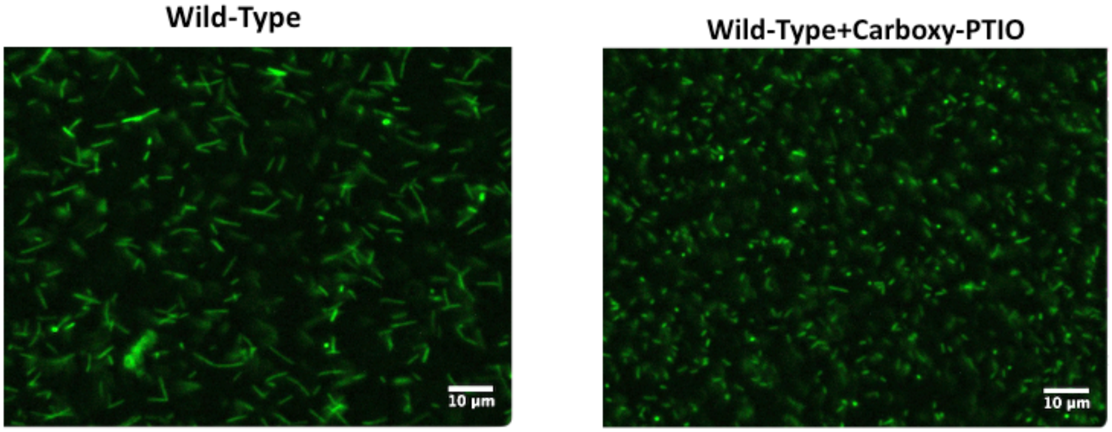


**Figure S4. The wild-type strain does not elongate during stationary phase in the absence of NO.** Micrographs of wild-type, after 16 h of anaerobic growth in LB, supplemented with 25 mM $NaNO_3$ and 2 mM carboxy-PTIO (a NO scavenger), are shown. Cells were fixed in 4% paraformaldehyde, stained with Syto9, and pipetted onto a microscope slide. Two independent cultures (biological replicates) were imaged in 3-5 random locations; representative images are shown. Loss of elongation in the wild-type strain suggests that elongation during stationary phase is due to accumulation of NO.

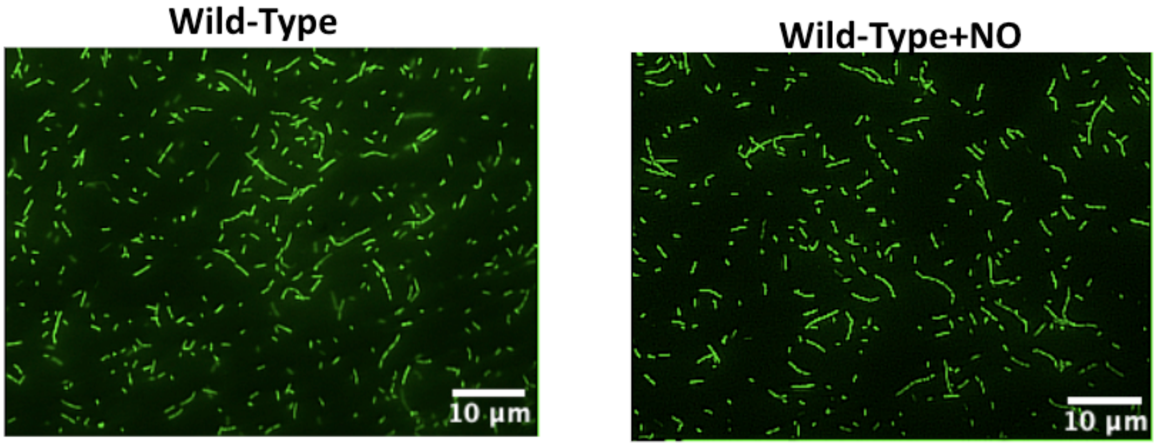


**Figure S5. Wild-type elongates during stationary phase anaerobically in the presence of exogenous NO.** Micrographs of wild-type, after 16 h of anaerobic growth in LB, supplemented with 25 mM $NaNO_3$ and 100 µM DETA-NONOate (a NO donor; ~ 100 nM NO), are shown. Cells were fixed in 4% paraformaldehyde, stained with Syto9, and pipetted onto a microscope slide. Three independent cultures (biological replicates) were imaged in 3-5 random locations; representative images are shown. At this timepoint, the wild-type strain elongates in both the presence and absence of exogenous NO, presumably because NO is accumulating already in the wild-type strain under these conditions, so additional addition of NO does not have an effect.

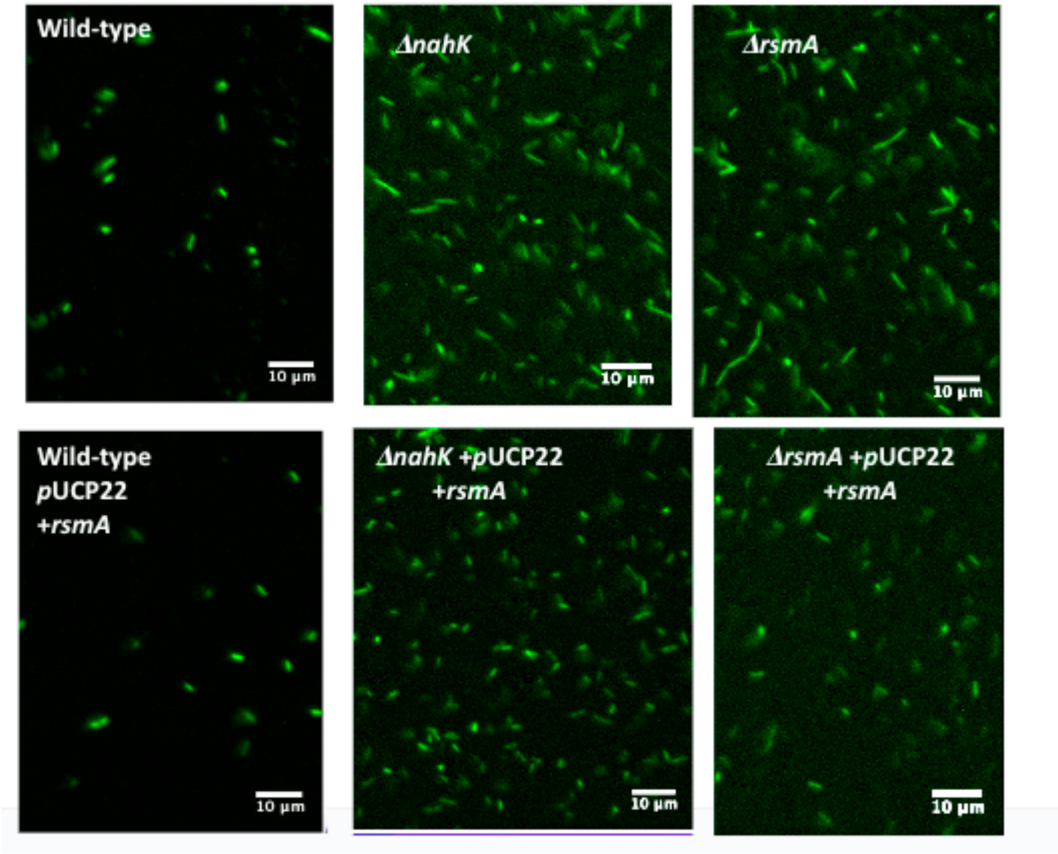


**Figure S6. Reduced *rsmA* levels in Δ*nahK* may contribute to its early elongation phenotype.** Micrographs of wild-type, Δ*nahK*, and Δ*rsmA* strains, with and without plasmid-based expression of *rsmA*, after 4 h of growth are shown. Bacteria were grown under anaerobic conditions in LB media supplemented with 25 mM $NaNO_3$ for 4 h. Cells were fixed in 4% paraformaldehyde, stained with Syto9, and pipetted onto a microscope slide. Three independent cultures (biological replicates) were imaged in 3-5 random locations; representative images are shown. Overexpression of *rsmA* in Δ*nahK* and Δ*rsmA* reduces elongation to near wild-type levels, suggesting low *rsmA* levels cause elongation in the Δ*nahK* and Δ*rsmA* strains.

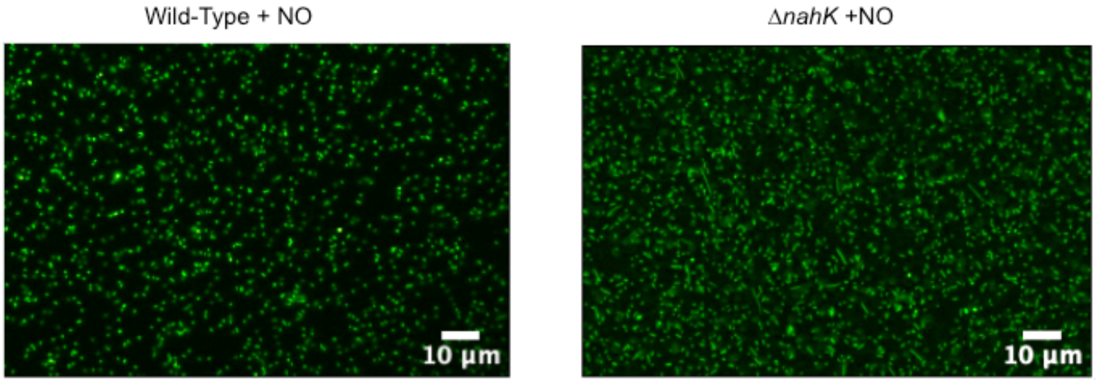


**Figure S7. Addition of exogenous NO to wild-type and Δ*nahK* strains grown aerobically does not cause elongation in stationary phase.** Micrographs of wild-type and Δ*nahK*, aerobically grown for 16 h the presence of 100 μM DETA-NONOate (~ 100 nM NO) are shown. Cells were fixed in 4% paraformaldehyde, stained with Syto9, and pipetted onto a microscope slide. Three independent cultures (biological replicates) were imaged in 3-5 random locations; representative images are shown. In aerobically grown cultures, addition of NO is insufficient to cause elongation in either strain.
